# Multivariate analysis of factors associated with first-pass success in blind placement of a post-pyloric feeding tube: a retrospective study

**DOI:** 10.1186/s40560-021-00577-1

**Published:** 2021-10-07

**Authors:** Kohei Kurisawa, Masashi Yokose, Hiroyuki Tanaka, Takahiro Mihara, Shunsuke Takaki, Takahisa Goto

**Affiliations:** 1 Department of Anesthesiology and Critical Care Medicine, Yokohama City University Graduate School of Medicine, 3-9 Fukuura, Kanazawa-ku, Yokohama, 236-0004 Japan; 2 Department of Health Data Science, Yokohama City University Graduate School of Data Science, Yokohama, Japan

**Keywords:** Blind placement, Enteral feeding tube, Intensive care unit, Post-pyloric enteral nutrition, Stomach, Greater curvature

## Abstract

**Background:**

Trans-jejunal nutrition via a post-pyloric enteral feeding tube has a low risk of aspiration or reflux; however, placement of the tube using the blind method can be difficult. Assistive devices, such as fluoroscopy or endoscopy, are useful but may not be suitable for patients with hemodynamic instability or severe respiratory failure. The aim of this study was to explore factors associated with first-pass success in the blind placement of post-pyloric enteral feeding tubes in critically ill patients.

**Methods:**

Data were obtained retrospectively from the medical records of adult patients who had a post-pyloric enteral feeding tube placed in the intensive care unit between January 1, 2012, and December 31, 2018. Logistic regression analysis was performed to assess the association between first-pass success and the independent variables. For logistic regression analysis, the following 13 variables were defined as independent variables: age, sex, height, fluid balance from baseline, use of sedatives, body position during the procedure, use of cardiac assist devices, use of prokinetic agents, presence or absence of intestinal peristalsis, postoperative cardiovascular surgery, use of renal replacement therapy, serum albumin levels, and position of the greater curvature of the stomach in relation to spinal levels L1 − L2.

**Results:**

Data obtained from 442 patients were analyzed. The first-pass success rate was 42.8% (*n* = 189). Logistic regression analysis demonstrated that the position of the greater curvature of the stomach cephalad to L1 − L2 was only associated with successful placement (odds ratio for first-pass success, 0.62; 95% confidence interval: 0.40 − 0.95).

**Conclusions:**

In critically ill patients, the position of the greater curvature of the stomach caudal to L1 − L2 may be associated with a lower first-pass success rate of the blind method for post-pyloric enteral feeding tube placement. Further studies are needed to verify our results because the position of the stomach was estimated by radiographs after enteral feeding tube placement.

*Trial registration:* University Hospital Medical Information Network Clinical Trials Registry (UMIN000036549; April 20, 2019).

## Background

Critically ill patients admitted to the intensive care unit (ICU) are a high-risk group for malnutrition, with a reported prevalence ranging from 38 to 78% [1]. Malnutrition is associated with muscle atrophy, prolonged ventilation, longer ICU stays, and increased risk of infection and mortality [2–5]. Enteral nutrition is superior to intravenous nutrition in terms of the incidence of infection [6, 7], length of hospital stay [8], and medical costs [9, 10]. Early initiation of enteral nutrition is also recommended to preserve endothelial cell structure and secretory function, and to maintain immunity [11–14]. Post-pyloric enteral nutrition decreases the incidence of respiratory complications compared to trans-gastric feeding [15, 16], and it is suitable for patients receiving sedatives or muscle relaxants, or those who cannot tolerate elevation of the head of the bed.

Methods of post-pyloric placement of enteral feeding tubes (EFTs) include endoscopy, fluoroscopy, ultrasound assistance, and electromagnetic guidance. However, EFTs can also be placed blindly without the aid of assistive devices. Fluoroscopy and endoscopy have higher success rates [17–19]; however, they may not be suitable for patients with hemodynamic instability or severe respiratory failure who cannot be transported outside the ICU. Blind placement at the bedside is commonly used in critically ill patients because it is easy, minimally invasive, and inexpensive. However, there is a considerable risk of failure in placing the tube in the correct position, which may lead to delays in enteral nutrition. To our knowledge, only a few studies have investigated the risk factors associated with blind placement difficulties [20].

In this study, we aimed to explore the factors associated with first-pass success of blind placement of post-pyloric EFTs.

## Methods

This retrospective observational study was conducted in accordance with the principles of the Declaration of Helsinki. The study design was approved by the Ethics Board of Yokohama City University Hospital, Yokohama, Japan (approval number: B181000027; November 22, 2018). The study was registered with the University Medical Information Network Clinical Trials Registry (UMIN000036549; April 19, 2019; principal investigator, Masashi Yokose) before data collection. The Ethics Board waived the requirement to obtain written informed consent owing to the retrospective nature of the study. Consecutive subjects aged ≥ 20 years who underwent blind placement of a post-pyloric EFT in the ICU of Yokohama City University Hospital from January 1, 2012, to December 31, 2018, were included in this study. The exclusion criteria were a preexisting EFT upon ICU admission, enteric fistula or gastrostomy, and a history of upper gastrointestinal surgery.

### Standard procedure for blind EFT placement

We used an EFT with a stylet (Kangaroo™ New Enteral Feeding Tube: Covidien Japan; Tokyo, Japan) in all patients. The size of the EFT was selected from 8 to 12 French according to the patient physique. The use of prokinetic agents was permitted when the attending physician felt it was clinically necessary. The right lateral position was selected when the patient’s condition permitted; however, this could be changed at the discretion of the physician performing EFT placement. The EFT was inserted through the nose or mouth and was advanced until the tip was presumed to be in the stomach (40 − 65 cm), which was confirmed by the sound of the gas over the stomach when 5–10 mL of air was forcibly injected through the tube. The tube was then slowly advanced a few centimeters at a time. Advancement of the tube was confirmed by releasing the hand after each push of the tube and ensuring that the tube stayed in position. If it was judged that the tube had not advanced, the tube was withdrawn about 5 cm and advanced again. To estimate the position of the EFT tip, air was forcibly injected through the tube to determine the strongest point of the sound. When the high-pitched sound was most audible in the patient’s right lateral abdomen, and the insertion length was approximately 25–30 cm from the point where it was determined to be in the stomach, 20 mL of water or air was injected through the EFT, and the tube was aspirated to confirm that nothing came back. Abdominal radiographs were taken to confirm that the tip of the EFT was in the jejunum. This standardized procedure at our institution, which was based on a previously published protocol with minor modifications [21], had already been established several years before the first participants for our study were admitted to our ICU. The procedure was taught among inexperienced or novice physicians as following steps: (1) physicians took the lecture using the document about this procedure by instructors who were specialists of critical care; (2) they observed the procedure performed by instructors; (3) in the presence of instructors, the educated physicians performed the procedure several times. Abdominal radiographs were evaluated by the medical team, including specialists who have much experience with post-pyloric placement, and the findings, including the position of the EFT tip, were documented in the medical record. The decision to start enteral nutrition via post-pyloric EFT was decided by a consensus within a medical team. The enteral nutrition via the post-pyloric route in our institution was used as the first choice, in principle.

### Data acquisition

**Fig. 1:**
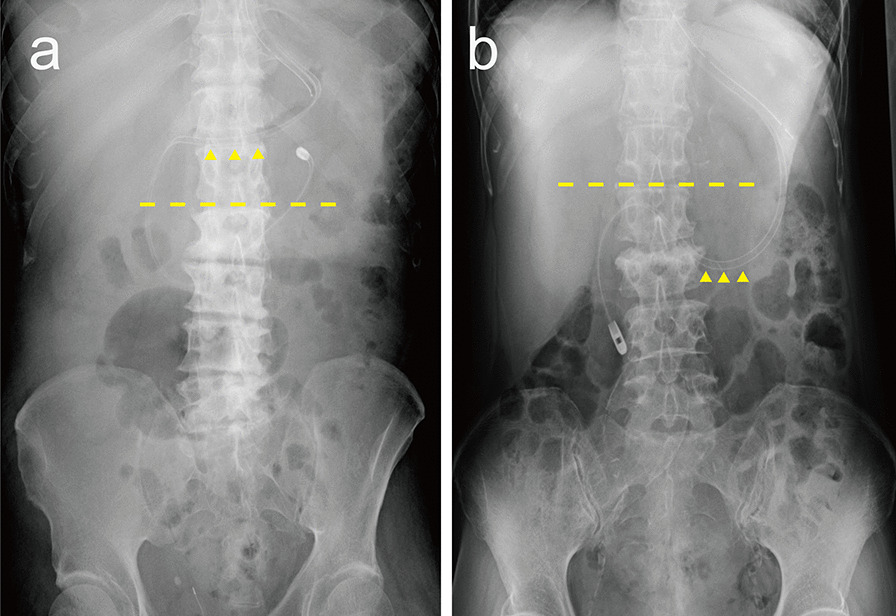
Examples of the determination of stomach position. The dashed line shows the spinal levels L1 − L2. Arrowheads show the enteral feeding tube at its lowest point in the stomach. **a** The position of the stomach is cephalad to L1 − L2. **b** The position of the stomach is caudal to L1 − L2

The following 21 variables were extracted from electronic medical records, as candidate predictors: (1) age; (2) sex; (3) height; (4) body mass index (BMI); (5) fluid balance from admission to the procedure; (6) presence or absence of intestinal peristaltic movement; (7) serum albumin levels; (8) body position during the procedure; (9) position of the stomach; (10) Sequential Organ Failure Assessment (SOFA) score at the time of the procedure; (11) postoperative cardiovascular surgery; (12) blood disorders (leukemia, myelodysplastic syndrome, or malignant lymphoma); (13) respiratory diseases (acute respiratory distress syndrome, pneumonia, or acute exacerbation of chronic obstructive pulmonary disease or pulmonary fibrosis); (14) concurrent diabetes mellitus; (15) use of prokinetic agents; (16) use of sedatives; (17) opioid dosage; (18) use of vasopressor agents; (19) use of cardiac assist devices (intra-aortic balloon pumping, extra-corporeal membrane oxygenation, or ventricular assist device); (20) use of renal replacement therapy, and (21) ICU experience of the physician performing the procedure. BMI was calculated based on the patient’s height and weight at ICU admission. Fluid balance was defined as an increase or decrease in body weight from ICU admission to the time of EFT placement. Albumin levels and SOFA scores were obtained from data collected closest to the time of EFT placement. The body position during the procedure was treated as a binary variable between right lateral and other positions. The position of the stomach was evaluated by the location of the greater curvature of the stomach relative to spinal level, as estimated by abdominal radiography after EFT placement; it was treated as a binary value of either cephalad or caudal to L1 − L2, which was the median value of our data (Fig. 1a, b). The amount of opioid used was converted to fentanyl dose per hour. The experience of the physician performing EFT placement was divided into four categories: junior residents, senior residents, fellows, and critical care specialists or supervisors. The presence or absence of in-hospital mortality, ICU length of stay, ventilator-free days (VFD), and EFT-related outcomes (days from ICU admission to the start of enteral nutrition and days from EFT placement to the start of enteral nutrition) were extracted from the medical records. VFD was defined as 28 days minus the number of days with mechanical ventilation via tracheal intubation after ICU admission.

### Outcomes

The primary outcome was the first-pass success rate of post-pyloric EFT placement. First-pass success was determined based on whether the EFT passed through the pylorus, as seen on the first abdominal radiograph after placement. If it was difficult to determine success or failure, it was comprehensively determined by two researchers who were involved in the research, referring to supplemental information such as descriptions in the medical records regarding the position of the EFT tip or the presence or absence of starting nutrition via EFT immediately after taking a first abdominal radiograph. The secondary outcomes were: (1) the correlation coefficient between the number of days from ICU admission to the start of enteral nutrition and the ICU length of stay; (2) the correlation between the number of days from EFT placement to the start of enteral nutrition and ICU length of stay, and (3) a description of patient outcomes.

### Sample size calculation

The reported success rate of blind placement of post-pyloric EFTs varies between 30 and 90% [22–25]. In this study, we assumed that the success rate was 60%, and 18 factors were selected as independent variables for logistic regression analysis. As the adopted number of negative events per variable was 10, the total number of negative events required was 180 (i.e., multiplying 18 variables by 10). Therefore, we calculated 450 as the minimum number of patients required.

### Statistical analyses

All data are expressed as median (interquartile range) or numbers (percentages), as appropriate. Logistic regression analysis was performed to assess the association between first-pass success and the independent variables. We selected variables with a *P* < 0.2 in the univariate analysis, and variables expected to be involved in the first-pass success rate based on the clinical perspective, as the independent variables. In the univariate analysis, the unpaired *t*-test, Mann–Whitney *U* test, or Fisher’s exact test was performed, as appropriate. All parameters were checked for multicollinearity. We performed complete data analysis to address missing values. Correlation coefficients for secondary outcomes were assessed using Spearman’s rank correlation. For all analyses, a two-tailed *P* < 0.05 was considered statistically significant. All statistical analyses were performed using Microsoft Excel 2016 (Microsoft; Redmond, WA, USA) and R software (version 3.3.2: R Foundation for Statistical Computing; Vienna, Austria).

## Results

**Fig. 2:**
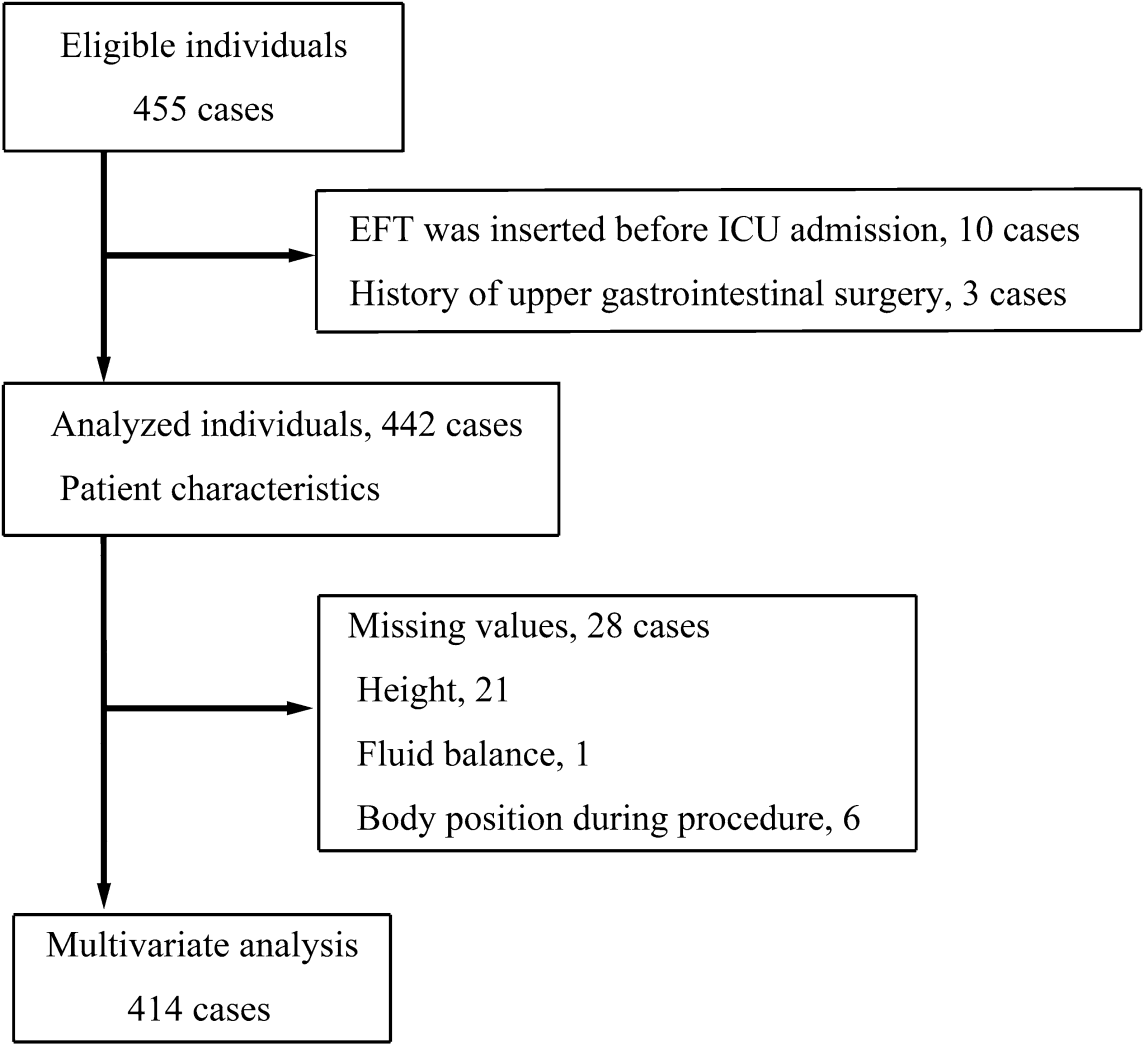
Study flowchart. *EFT* enteral feeding tube; *ICU* intensive care unit

A total of 455 patients were included in this study. After 13 patients were excluded (10 patients, EFT was inserted before ICU admission; three patients, a history of upper gastrointestinal surgery), we analyzed data from 442 patients who met the inclusion criteria (Fig. 2). Table 1 shows the patient characteristics. The median age and SOFA score were 68 (57 − 76) years and 10 (7 − 13), respectively. The percentage of men was 60%. The first-pass success rate of blind placement of a post-pyloric EFT was 42.8% (*n* = 189). The median ICU length of stay and VFD was 8 (6 − 14) days and 21 (6 − 24) days, respectively. The number of patients who died in the ICU was 35 (8%) (Table 2).

**Table 1:** Patient characteristics and results of the univariate analysis

Characteristic	Overall (*n* = 442)	Success (*n* = 189)	Failure (*n* = 253)	*P*-value
Age (years)	68 (57 − 76)	67 (54 − 75)	68 (59 − 77)	0.19
Sex, *n* (%)				
Male	265 (60)	103 (23)	162 (37)	0.049
Female	177 (40)	86 (19)	91 (21)	
Height (cm)	162 (154 − 168)	160 (153 − 168)	162 (155 − 168)	0.20
Weight (kg)	58 (48 − 66)	58 (48 − 65)	58 (50 − 67)	0.45
Body mass index (kg/m^2^)	22.4 (19.8 − 24.9)	22.4 (19.8 − 24.7)	22.4 (19.8 − 25.2)	0.92
Fluid balance from baseline (kg)	2.3 (0.4 − 4.8)	2.5 (0.6 − 0.9)	2.1 (0.2 − 4.6)	0.30
SOFA score	10 (7 − 13)	10 (7 − 12)	10 (7 − 13)	0.87
Serum albumin (g/dL)	2.7 (2.3 − 3.1)	2.7 (2.2 − 3.1)	2.8 (2.3 − 3.2)	0.12
Position of the stomach	L1 − L2 (T12 − L1 to L2 − L3)	L1 − L2 (T12 − L1 to L2 − L3)	L1 − L2 (T12 − L1 to L2 − L3)	0.06
Use of sedatives,* n* (%)				
No	49 (11)	25 (13)	24 (9)	0.22
Yes	393 (89)	164 (87)	229 (91)	
Use of a vasopressor, *n* (%)	307 (69)	132 (70)	175 (69)	0.92
Fentanyl dose (µg/hour)	20 (10 − 20)	20 (0 − 20)	20 (10 − 25)	0.38
Right lateral position, *n* (%)	169 (38)	75 (40)	94 (37)	0.69
Use of a prokinetic agent, *n* (%)	22 (5)	11 (6)	11 (4)	0.51
Presence of intestinal peristalsis,* n* (%)	116 (26)	58 (30)	58 (23)	0.08
Degree of experience,* n* (%)				
Junior resident	84 (19)	34 (18)	50 (20)	0.45
Senior resident	47 (11)	23 (12)	24 (10)	
Fellow	107 (24)	41 (22)	66 (26)	
Specialist	21 (5)	10 (5)	11 (4)	
Missing values	183 (41)	81 (43)	102 (40)	
Diabetes mellitus, *n* (%)	92 (21)	36 (19)	56 (22)	0.48
Renal replacement therapy, *n* (%)	50 (11)	16 (8)	34 (13)	0.13
Use of cardiac assist devices, *n* (%)	36 (8)	13 (7)	23 (9)	0.48
Post-cardiac surgery, *n* (%)	180 (41)	69 (37)	111 (44)	0.14
Hematology, *n* (%)	43 (10)	20 (11)	23 (9)	0.63
Respiratory disease, *n* (%)	114 (26)	52 (28)	62 (25)	0.51

*Body mass index* was calculated based on the height and weight at intensive care unit (ICU) admission. *Cardiac assist devices* included intra-aortic balloon pumping, extra-corporeal membrane oxygenation, and ventricular assist devices. *Fluid balance* was defined as an increase or decrease in body weight from ICU admission to enteral feeding tube placement. *Hematology* included patients with blood disorders, such as leukemia, myelodysplastic syndrome, and malignant lymphoma. *Post-cardiac surgery* included patients admitted to the ICU for postoperative management of cardiovascular surgery. *Respiratory disease* included patients with acute respiratory distress syndrome, pneumonia, and acute exacerbation of chronic obstructive pulmonary disease or pulmonary fibrosis. *Position of the stomach* was defined as the position of the greater curvature of the stomach caudal or cephalad to L1–L2, as estimated by abdominal radiography. *Weight* was defined as body weight before ICU admission. All values are expressed as number (percentage) or median (interquartile range). *SOFA* Sequential Organ Failure Assessment

**Table 2:** Summary of outcome data

Outcome	Overall (*n* = 442)	Success (*n* = 189)	Failure (*n* = 253)
Death in the ICU, *n* (%)	35 (8)	18 (4)	17 (4)
Death in the hospital, *n* (%)	100 (23)	42 (10)	58 (13)
Death after ICU admission (days)	27 (13 − 60)	34 (14 − 70)	23 (12 − 42)
Ventilator-free days	21 (6 − 24)	20 (5 − 24)	21 (6 − 24)
Time from EFT placement to the start of enteral feeding (days)	1 (0 − 2)	0 (0 − 1)	1 (1 − 2)
Time from ICU admission to the start of enteral feeding (days)	2 (1 − 4)	2 (1 − 3)	3 (2 − 4)
ICU length of stay (days)	8 (6 − 14)	8 (6 − 15)	8 (5 − 13)

*Death in the hospital* was defined as the number of patients who died in the ICU or on the ward. *Ventilator-free days* were calculated as 28 days minus the number of days without mechanical ventilation via tracheal intubation. All values are expressed as number (percentage) or median (interquartile range). *EFT* enteral feeding tube, *ICU* intensive care unit

The eight variables with *P* < 0.2 in the univariate analysis were: age, sex, height, serum albumin levels, position of the stomach, presence of intestinal peristaltic movement, renal replacement therapy, and post-cardiovascular surgery (Table 1). In addition, five factors were selected as variables expected to be involved in the first-pass success rate based on the clinical perspective: body position during placement, use of prokinetic agents, fluid balance, use of cardiac assist devices, and use of sedatives. Because multicollinearity was not found in these 13 variables, all variables were included in the final analysis. Twenty-eight cases with missing values (21 cases, height; one case, fluid balance; and six cases, body position during EFT placement) were excluded from the final analysis. The results of the logistic regression analysis with these 13 factors as independent variables are shown in Table 3.

**Table 3:** Multivariate analysis of the first-pass success rate of enteral feeding tube placement

Variable	Odds ratio	95% CI	*P*-value
Age (each 10-year increment)	0.91	0.78 − 1.06	0.22
Sex (female)	1.32	0.74 − 2.37	0.35
Height (each 10-cm increment)	0.91	0.68 − 1.23	0.55
Fluid balance (each 1-kg increment)	1.04	0.98 − 1.10	0.18
Serum albumin level (each 1-g/dL increment)	0.74	0.50 − 1.07	0.11
Position of the stomach (caudal to L1 − L2)	0.61	0.40 − 0.94	0.03
Use of sedatives	0.80	0.42 − 1.55	0.51
Right lateral body position	1.01	0.66 − 1.53	0.97
Use of prokinetic agents	1.20	0.47 − 3.07	0.70
Presence of intestinal peristaltic movement	0.75	0.47 − 1.20	0.23
Renal replacement therapy	0.52	0.26 − 1.05	0.07
Use of cardiac assist devices	0.86	0.39 − 2.01	0.77
Post-cardiovascular surgery	0.96	0.58 − 1.60	0.87

Factors with an odds ratio > 1.0 are associated with successful EFT placement. *CI* confidence interval

A stomach position caudal to L1 − L2 was associated with a lower first-pass success rate (adjusted odds ratio, 0.61; 95% confidence interval: 0.40 − 0.94*; P* = 0.03). The number of days from ICU admission to the start of enteral nutrition was 2 (1 − 4) days. The correlation coefficient between the number of days from ICU admission to the start of enteral nutrition and ICU length of stay was 0.22 (*P* < 0.001). The median number of days from ICU admission to the start of enteral nutrition in patients with first-pass success was lower than that in those without first-pass success. The absolute difference was 1 day (first-pass success *vs.* failure: 2 [1 − 3] *vs*. 3 [2 − 4] days) (Table 2). The correlation coefficient between the number of days from EFT placement to the start of enteral nutrition and ICU length of stay was 0.15 (*P* < 0.001). The median ICU length of stay was 8 days in both the first-pass success and failure groups (Table 2).

## Discussion

We demonstrated that a position of the greater curvature of the stomach lower than spinal level L1 − L2 may be associated with a lower first-pass success rate of blind placement of a post-pyloric EFT in critically ill patients. Notably, the stomach position was evaluated using abdominal radiography after EFT placement as part of the clinical procedures to confirm correct EFT placement. However, because the position of the stomach was estimated after EFT placement, whether the stomach position before EFT placement is associated with the success of blind tube placement is unknown.

Our study was not designed to elucidate the mechanism(s) underlying our findings; however, several possibilities warrant consideration. First, a more caudal position of the greater curvature of the stomach results in a more angulated path from the gastric inlet (the cardia) to the outlet (the pylorus), which is more difficult for the EFT to travel along. Second, a more caudal position of the greater curvature of the stomach may indicate greater extensibility of the stomach. Gastric extensibility varies greatly among individuals [26, 27]. A more extensible stomach may have absorbed the incremental advancement of the EFT instead of guiding it in the direction of the pylorus, resulting in failure of the tube to pass through the pylorus. Third, a gastric extension may also have extended the length of the EFT required for pyloric passage. Therefore, there may have been cases where the EFT did not reach the pylorus because the physicians may have been hesitant to insert the EFT beyond the depth of their empirical predictions.

The reported success rate of post-pyloric EFT placement varies greatly [22–25, 28, 29]. Our success rate was 42.8%, which is unfavorable compared to previous studies. This may be explained by the subtle differences in detailed placement procedures/protocols or by differences in the study populations, such as the inclusion or exclusion of patients with circulatory instability [28] or a high BMI [29]. Our study population, which included these patients, may reveal a success rate in a patient population with higher severity. Conversely, in our retrospective study, the lack of assessment of the adherence rate to our institutional protocol for EFT placement procedures may be a disadvantage for generalization, because a lower adherence rate may be associated with a lower success rate. In our ICU, care was taken to ensure the quality of the placement procedures when performed by inexperienced staff by having more experienced staff supervise.

A recent study suggested that several factors, including the severity of the participants’ condition (Acute Physiology and Chronic Health Evaluation II score ≥ 20, SOFA score ≥ 12, use of vasopressor, and patients with mechanical ventilation), patients with neurological diseases, use of continuous renal replacement therapy, and impaired gastrointestinal function, may be associated with the success rate of blind tube placement [20]. Elucidation of risk factors may provide additional information for the strategy of nutrition therapy in critically ill patients. However, further evidence will need to be accumulated because the settings in the studies reported previously are limited.

Our study could not elucidate the effect of prokinetic agents due to the wide confidence interval caused by the small number of patients who received them. A previous meta-analysis showed that the use of prokinetics in patients without intestinal peristalsis promoted successful EFT placement [30]. However, the Cochrane review in 2015 argued that a higher-quality, large randomized controlled study is necessary to examine this further [31].

Our study had several limitations. First, this was a retrospective, single-center study and, therefore, could not be adjusted for unknown confounders, and external validity may be limited. Second, the confidence intervals of odds ratios for several variables were wide, partly because of the small number of positive cases. Third, the experience levels of the physicians performing the EFT placements could not be evaluated because there was too much missing data. It was difficult to quantitatively evaluate the experience level of the physicians with high accuracy in retrospective data obtained from the medical record. We excluded this factor from the final analysis because: first, we were concerned that the reduction in the number of cases might have made it difficult to achieve our goal of a comprehensive search for associated factors. Second, in our ICU, the quality of the procedures was ensured by the supervision of senior physicians. Additional logistic regression analysis, which was a sensitivity analysis including experience levels of the physician, confirmed that the position of the stomach, which was the main result, had a similar direction of effect (data not shown). Finally, first-pass success or failure of EFT placement which was our primary outcome, might include the bias because it was estimated by the investigators associated with the study.

In this study, the position of the stomach was estimated after, but not before, EFT placement. Therefore, our results cannot be used to identify predictive factors before EFT placement. The main objective of this study was to generate hypotheses through a comprehensive investigation of factors associated with first-pass success. Therefore, we used the abdominal radiographs after EFT placement simply as an index of the position of the stomach because it is our routine clinical practice to confirm the position of the EFT by abdominal radiographs after the placement. Because our clinical practice does not require pre-placement abdominal radiographs or computed tomography, we were afraid that incorporation of the pre-placement abdominal radiographic examination into our study design would reduce the number of cases that could be enrolled, leading to insufficient statistical power for multivariate analysis. Further studies are needed to determine whether a lower stomach position on abdominal radiograph or computed tomography before EFT placement can predict the difficulty of EFT placement.

## Conclusions

The greater curvature of the stomach caudal to spinal level L1 − L2 may be associated with the first-pass failure of post-pyloric EFT placement. Further studies with larger numbers of patients are needed to verify whether the lower stomach position before EFT placement can predict the difficulty of EFT placement.

## Data Availability

The datasets used and/or analyzed during the current study are available from the corresponding author on reasonable request.

## Abbreviations

BMI: Body mass index
CI: Confidence interval
EFT: Enteral feeding tube
ICU: Intensive care unit
SOFA: Sequential Organ Failure Assessment
VFD: Ventilator-free days
